# Biota and Biomolecules in Extreme Environments on Earth: Implications for Life Detection on Mars

**DOI:** 10.3390/life4040535

**Published:** 2014-10-13

**Authors:** Joost W. Aerts, Wilfred F.M. Röling, Andreas Elsaesser, Pascale Ehrenfreund

**Affiliations:** 1 Molecular Cell Physiology, Faculty of Earth and Life Sciences, VU University Amsterdam, de Boelelaan 1085, 1081 HV Amsterdam, The Netherlands; E-Mail: wilfred.roling@vu.nl; 2 Leiden Observatory, Leiden University, P.O. Box 9513, NL-2300 RA Leiden, The Netherlands; E-Mails: a.elsaesser@umail.leidenuniv.nl (A.E.); pehren@gwu.edu (P.E.); 3 Space Policy Institute, George Washington University, Washington, DC 20052, USA

**Keywords:** biomarkers, Mars, minerals, adsorption, extreme environments, life detection, extraction techniques, origin of life

## Abstract

The three main requirements for life as we know it are the presence of organic compounds, liquid water, and free energy. Several groups of organic compounds (e.g., amino acids, nucleobases, lipids) occur in all life forms on Earth and are used as diagnostic molecules, *i.e.*, biomarkers, for the characterization of extant or extinct life. Due to their indispensability for life on Earth, these biomarkers are also prime targets in the search for life on Mars. Biomarkers degrade over time; *in situ* environmental conditions influence the preservation of those molecules. Nonetheless, upon shielding (e.g., by mineral surfaces), particular biomarkers can persist for billions of years, making them of vital importance in answering questions about the origins and limits of life on early Earth and Mars. The search for organic material and biosignatures on Mars is particularly challenging due to the hostile environment and its effect on organic compounds near the surface. In support of life detection on Mars, it is crucial to investigate analogue environments on Earth that resemble best past and present Mars conditions. Terrestrial extreme environments offer a rich source of information allowing us to determine how extreme conditions affect life and molecules associated with it. Extremophilic organisms have adapted to the most stunning conditions on Earth in environments with often unique geological and chemical features. One challenge in detecting biomarkers is to optimize extraction, since organic molecules can be low in abundance and can strongly adsorb to mineral surfaces. Methods and analytical tools in the field of life science are continuously improving. Amplification methods are very useful for the detection of low concentrations of genomic material but most other organic molecules are not prone to amplification methods. Therefore, a great deal depends on the extraction efficiency. The questions “what to look for”, “where to look”, and “how to look for it” require more of our attention to ensure the success of future life detection missions on Mars.

## 1. Introduction

It has been hypothesized that life will form and evolve whenever the energetic, chemical, and geological conditions are met. Organic molecules, typical for life as we know it, are relatively easily formed and can be polymerized into larger molecules under the right chemical conditions [1,2]. Urey and Miller revealed the abiotic formation of amino acids and related compounds in their famous 1953 experiment [3]. This experiment was recently repeated with modern analytical techniques, with similar results [4]. In another experiment, Levy *et al.* [5] found that amino acids were produced abiotically in a frozen NH_4_CH solution while Martins *et al.* [6] showed the formation of several amino acids through a process called impact shock synthesis. Hydrocarbons can be synthesized by chemical reactions simulating high-pressure/temperature conditions (Fischer-Tropsch reactions) [7,8,9], and nucleobases have been shown to form under simulated prebiotic conditions [10,11,12], with formamide (HCONH_2_) as the precursor molecule, which may have been available on the early Earth [12]. These findings, also taking into account the detection of a wide variety of biologically relevant molecules in meteorites [13], suggest that the building blocks of life as we know it are abundant throughout our solar system, which has intensified the search for life beyond Earth. However, when searching for molecular traces of life on other worlds, such as Mars, great care must be taken to distinguish between biotic and abiotic origin. Biologically produced or altered molecules hence have been termed *biomarkers* and typically possess specific signatures that link them to biotic origin. Current and future life detection missions to Mars express a strong focus towards detecting those biomarkers as evidence for extant or extinct life [14].

**Figure 1:**
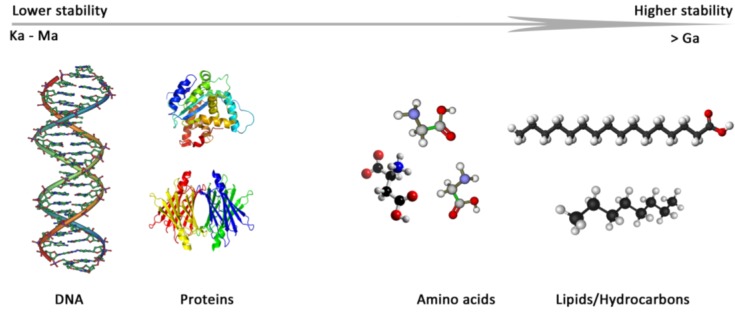
The preservation potential of several biomarkers in Ka (thousand years) to Ga (billion years). Modified from Martins *et al.* [37].

Early conditions on Mars, during the first billion years after planetary formation, may have allowed life to develop and remnants of it could have been preserved within protected niches [15]. Current conditions on Mars include extreme aridity, freezing temperatures (average −60 °C) and high UV-flux, and are damaging to living organisms and their organic molecules, decreasing the chances for life to be present [16,17]. Extreme environments on Earth are useful for astrobiologists since they often display environmental and geological parallels with current and past Martian conditions. Investigating those environments, and the effects they impose on life on Earth and the preservation of its associated biomarkers, has already contributed greatly to the search for life on Mars and other planets. Even in the most extreme environments on modern-day Earth, life forms are identified [18,19]. Recent findings [20,21,22] reveal that life can thrive in environments we thought previously uninhabitable, suggesting we have not yet encountered the limits of life on our own planet. Of special interest are subsurface environments where life has been cut off from sunlight, but instead manages to thrive solely on chemosynthesis, as observed in Movile Cave, Romania [23]. The identification of ancient biomarkers on Earth [24,25,26,27,28] suggests strong preservation potential for a subset of biomarkers under the right conditions, however large differences between preservation potential exist among biomarkers (see Figure 1 for a schematic representation of biomarker stability). Such observations provide important information on which biomarkers to look for and where to look for them on Mars.

The success of life detection depends, besides the obvious necessity of an organic inventory, on a trade-off between specificity, sensitivity and extraction efficiency of the applied techniques. Techniques in the field are continuously improving and molecules can be detected in the range between parts per billion and part per trillion (ppb-ppt) [29]. This sensitivity is important since organic molecules are often strongly adsorbed to mineral surfaces, decreasing extraction and, thus, the chance for detection considerably [30,31]. However, minerals are considered major targets for future missions to Mars due to their importance in biological processes. Polymerization of small molecules (e.g., amino acids) occurs on mineral surfaces [32], linking them to the origin of life [33]. Minerals also have nutritional value for microorganisms and it is becoming increasingly clear that specific minerals are selected by distinct microbial populations due to the absence/presence of certain trace elements [34,35]. In addition, the organics-preserving effects observed for minerals makes them prime targets for the search for life on Mars [36]. It is therefore of great importance that extraction of biomarkers from their environmental context is as efficient as possible.

Here, we review current approaches of sensitive life detection. Special attention will be given to a range of biomolecules indicative for life, such as DNA, amino acids, lipids, and their diagenetic breakdown products, and how useful they are as biomarkers based on their general properties, like preservation potential, specificity, and extractability.

We describe recent findings of terrestrial life in extreme environments and how these results may help in determining the most promising landing sites on Mars for future life detection missions. We have included the analysis of carbonaceous meteorites since they represent a unique extraterrestrial source of carbon compounds, which may have seeded planets in our solar system with organics through impact events. We also describe techniques currently in use for life detection on Earth and Mars and how techniques in development may improve sensitivity and efficiency of life detection. We conclude with a discussion concerning the implications for life detection on future planetary missions to Mars.

## 2. Biomarkers: What to Look for?

In the search for traces of extant or extinct life, a wide variety of diagnostic biomarkers can be targeted. Factors like intrinsic stability and specificity determine how useful a biomarker can be for the intended purpose: the detection of extant or extinct life. The necessity of a protected environment is a perquisite for any type of biomarker to be preserved over longer geological timescales (Figure 1) but variation in molecular stability is also an important parameter to consider. The ideal approach would be to scan for biomolecules with a relatively high lifespan that indicate unambiguously biogenic origin. Parnell *et al.* [14] devised a priority list of potentially interesting diagnostic molecules to be targeted for life detection on Mars. The next three sections will focus on several of these biomarker classes and describe their potential as target molecules.

### 2.1. Deoxyribonucleic Acid (DNA)

The detection of extraterrestrial DNA would provide unequivocal proof of the presence of extant life, or at least its presence in the recent past on Mars. All terrestrial life forms store their hereditary information in DNA [38]. Obviously, life on other planets does not have to be based on an exact replica of terrestrial DNA, and common gene sequences primed for on Earth may not be present in extraterrestrial DNA [39]. Life on other worlds may not even utilize DNA-like molecules at all to store hereditary information. Nonetheless, within our solar system lies the potential to spread material from one planet to another through impact or expulsion events, theoretically paving way for an interplanetary ancestor of all life in our solar system, or at least for Earth and Mars where conditions once were very similar. Hence, finding DNA-based life on Mars is plausible.

The opposite strands of the double helix of terrestrial DNA are held together via double and triple hydrogen bonds between the nucleobases, and the sugar-phosphate backbone maintains the side-by-side position of the bases. The negative charge of the phosphate backbone makes it prone to adsorption by clay-rich minerals through ion-exchange interactions, which may complicate extraction [40].

In a recent study, focusing on the adsorption behavior of a variety of pure minerals, it was shown that clay minerals adsorb up to 99% of DNA, which could not be recovered [30]. On the other hand, the protective nature of certain minerals can shield the DNA from oxidizing conditions, enzymatic degradation and electromagnetic radiation such as ultraviolet (UV) radiation and X-rays [41,42,43], which would otherwise quickly destroy DNA molecules.

The timescales that DNA can persist in the fossil record are still debated although lifetimes of at least several tens of thousands to a hundred thousand years [44,45] are generally accepted. Recently, Orlando *et al.* [28] raised the bar by isolating and sequencing an intact horse genome dating 700,000 years back. Claims of ancient DNA dating millions of years, such as the isolation of a 250 million years old bacterium from halite deposits [46] have endured major criticism and are often assumed to be the result of flawed experiments or contamination [27]. Nonetheless, there are indications that certain conditions could improve the conservation of ancient DNA. Halite crystals, amber depositions, permafrost and marine sediments all have potential for the long-term preservation of DNA [45,47]. Even more so, the low temperatures and desiccated conditions on Mars may help to preserve DNA molecules much better than on Earth [48], making it still an attractive target.

The Polymerase-Chain-Reaction (PCR) technique enables to amplify minute amounts of DNA [49]. This is an enormous advantage for life detection in samples with low biomass since it facilitates detection of life that would remain undetected with other methods. However, the downside of the widely used PCR amplification technique is the risk of contamination, especially when working with low biomass samples.

The sensitivity of DNA *vis à vis* destructive environmental factors and the relatively short lifetime of the molecule make it less useful as a biomarker for extinct life. Nonetheless, the availability of amplification techniques and the undisputed role of DNA in biological evolution make it a tempting biomarker when looking for traces of extant or recently extinct life. A wide variety of nucleobases has been detected in carbonaceous meteorites [50,51] and the existence of nucleic acids with alternative backbones or nucleobases in extraterrestrial life is not unlikely [52]. The detection of nucleobases in meteorites indicates that fundamental building blocks of complex biopolymers are present beyond Earth and could potentially be incorporated in the evolution of life on other worlds.

### 2.2. Lipids 

All known living organisms possess lipid membranes. Membranes provide compartmentalization, protection and selective passage of molecules and ions, allowing for energy transduction. Lipids have high resistance to enzymatic degradation compared to proteins or nucleic acids [48], an important trait for these molecules since they are in direct contact with the external environment of the cell. Membrane components therefore would represent suitable indicators of past or present life. Unicellular life forms can be subdivided into archaea, bacteria, and eukaryotes, all exhibiting different types of lipids that contribute to their membrane integrity and regulation of their fluidity in response to physiochemical conditions like temperature or pH (Table 1) [48,53].

**Table 1:** Characteristic membrane molecules influencing membrane integrity.

Domain	Dominant Membrane molecule	Molecule build-up/adaptations	Typical chain length
Archaea	Isoprenoids	5-carbon isoprene unit incorporation, unsaturated branched side chains	20 carbon atoms
Bacteria	Fatty acids, Hopanoids	2-carbon acetyl incorporation, unsaturated *cis*-double bonds, addition of methyl groups	14–18 carbon atoms
Eukarya	Fatty acids, Steroids	2-carbon acetyl incorporation, unsaturated *cis*-double bonds	14–18 carbon atoms

**Figure 2:**
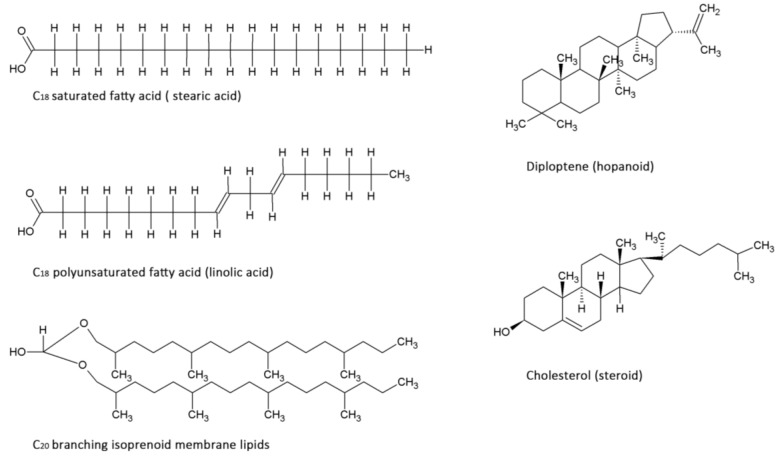
Examples of types of membrane lipid molecules that are used as diagnostic biomarkers.

Achaean membranes typically consist of a double layer of repeating 5-carbon isoprene units resulting in branched hydrocarbon chains (Figure 2). Since abiotic processes are not known to produce these molecules, they are strong indicators of life. Fatty acids, molecules primarily consisting of repeating 2-carbon acetyl units, are characteristic for bacterial and eukaryotic cell membranes (Figure 2). Bacteria typically incorporate hopanoids (Figure 2) whereas eukaryotes use steroids (Figure 2) for extra regulation of membrane fluidity [54]. Most lipid molecules undergo enzymatic changes that cannot be produced by non-biogenic reactions [55]. Variations in the lipids regulate the stability of the cell membrane, allowing adaptation to a wide variety of environmental conditions [56]. Examples are the enzymatic incorporation of *cis*-double bonds in fatty acids and incorporation of methyl groups by bacteria, both resulting in more membrane fluidity [57,58] (Figure 2). Additionally, increasing alkyl-chain length and incorporation of membrane spanning lipids are means for microorganisms to regulate their membrane fluidity [54]. These specific adaptations and characteristics of membrane molecules can be preserved for billions of years if the molecules are converted to stable hydrocarbons through diagenetic breakdown.

Due to the complexity and variability of processes involved in the diagenesis of a molecule, a multitude of intermediates and end products can arise over time from the same molecular precursor compound [59]. Through known chemical processes during diagenesis, such as reduction and oxidation, transformed hydrocarbons can often be traced back to their parent compound and thus its phylogenetic lineage [60]. A multitude of lipids and their hydrocarbon derivatives have been studied to identify ancient terrestrial life. Since fatty acids are major membrane components used in two of the three domains of life and because they show a high stability for extended periods of time [61], they represent excellent biomarkers for extant, but more importantly, extinct life. Polyunsaturated fatty acids (Figure 2) have been detected in ancient sediments and could be traced back to biogenic origin [62,63]. Pythane, a stabilizing component of Achaean membranes, including those of methanogens [64], has been extracted from ancient fossil rich subsurface sediments [24], hereby confirming its high preservation potential and diagnostic power as a biomarker for extinct life [14]. 2a-methylhopanes and steranes, which are representatives for cyanobacteria and eukaryotes, respectively, have been extracted from 2.7 billion-year old shales from the Pilbara Craton [24].

In addition to the biologically produced lipids and their hydrocarbon counterparts, a multitude of inorganically derived hydrocarbons exist that are particularly present in meteorites [65]. Polyaromatic hydrocarbons (PAHs) comprise a subgroup of those in carbonaceous chondrites. PAHs are non-biogenic in origin, and as such important indicators of carbon chemistry in outer space rather than biomarkers [66]. It has been suggested that these PAHs could be precursor molecules for biological processes [67]. Non-biogenically derived hydrocarbons often show extreme structural diversity, branched side chains and a decrease in abundance with increasing carbon number [68]. The random diversity of these abiotic hydrocarbons allows us to distinguish them from biogenic hydrocarbons, which possess specific structural and chemical characteristics. An attractive approach is to look for “deviations from the mean”. The odd-even pattern distribution of hydrocarbon mixtures is a well-established method to determine the contribution of a biological source to a hydrocarbon mixture [69]. During fatty acid biosynthesis, two carbon atoms are typically added at a time, while in abiotic monocarboxylic acid synthesis one carbon atom is added (or removed) at a time. This results in distinguishable patterns in which an excess of even numbered fatty acids of higher chain length points to biogenic origin.

In summary, lipids and their fossil counterparts are very stable and are in many cases selectively represented in the domains of life on Earth. Due to their hydrophobic properties they are not readily extractable by polar solvents and thus organic solvents are used when extracting lipids from a soil.

### 2.3. Amino Acids

Amino acids are among the most widespread biomolecules on Earth. Currently, more than 500 different amino acids have been identified [70]. Of special interest are the α-amino acids that have both the amine and carboxylic acid group attached to the first carbon (α) atom and have an organic substituent as the functional side chain. These amino acids include the 22 proteinogenic amino acids (Table 2) which are linked together by the cellular protein translating machinery into peptides resulting in the formation of proteins. Differences between these amino acids are largely responsible for the varying interaction with their surroundings. This may result in different extraction yields when recovering them from soils.

Amino acids contain both acid and base groups, giving them their zwitterionic properties [71], while the distinctive side chain of each amino acid is largely responsible for the variations in charge and polarity observed among amino acids. The charge of amino acids is affected by solution pH. Amino acid charge equals zero if the solution pH equals the isoelectric point (pI), which results from the amino acid side group. The adsorption of amino acids to mineral surfaces is strongly influenced by electrostatic interactions. The net charge of mineral surfaces is a function of pH. The net charge is zero when the solution’s pH is equal to the “point of zero charge” (pH_pzc)_. When the solution’s pH is lower than the pH_pzc_, the net surface charge of the mineral is positive, while a solution’s pH higher than the pH_pzc_ induces a negative net surface charge of the mineral. Interactions between minerals and amino acids are strongly influenced by those parameters and since all amino acids differ in pI and minerals all have distinct pH_pzc_ this results in complex interactions. However, a general tendency is seen where opposing pH_pzc_ and pI result in maximum adsorption [72]. Other factors also contribute significantly to adsorption [32,73], such as the distribution of positive and negative charges on specific mineral surfaces [72].

**Figure 3:**
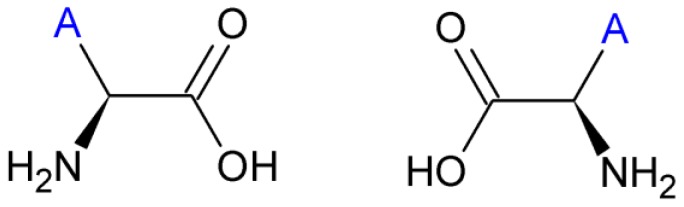
The principle of amino acid chirality. “A” depicts the side chain.

The proteinogenic amino acids, except glycine, display isomerism resulting in the existence of two distinct enantiomers, (levorotatory) l- and (dextrorotatory) d- amino acids (Figure 3). Under abiotic conditions, amino acid mixtures turn racemic over time. Because almost all terrestrial life uses amino acids in the l-formation (only a few cases of d-amino acids in biology are known), deviations from racemic mixtures are commonly used as proof of biological origin [74]. However, selective adsorption of d- or l- amino acids by minerals, such as calcite [75], could potentially complicate interpretation. Another inducer of homochirality is circular polarized ultraviolet light [76], which may explain excesses of l-amino acids in the Murchisson meteorite [77]. In contrast, *Glavin and Dworkin* [78] proposed that extended aqueous alteration in meteorite parent bodies is primarily responsible for the observed enantiomeric excesses (ee) observed, stating that an initial small asymmetry could have been amplified over an extended period of time. In favor of this proposed mechanism, Steendam *et al.* [79] showed complete deracemization of amino acids towards either enantiomer end state with equal probability, by increasing attrition during Viedma ripening. These abiotic processes should be considered when using homochirality as an exclusive indicator of biogenic origin.

**Table 2:** Amino acid structure and side chain characteristics at neutral pH.

Amino Acid	Side chain properties	Chemical structure	Amino Acid	Side chain properties	Chemical structure
Alanine	Hydrophobic side chain	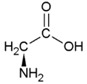	Serine	Polar, uncharged side chain	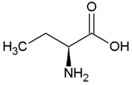
Valine	Hydrophobic side chain	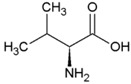	Threonine	Polar, uncharged side chain	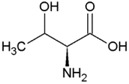
Leucine	Hydrophobic side chain	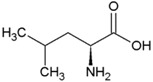	Asparagine	Polar, uncharged side chain	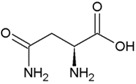
Isoleucine	Hydrophobic side chain	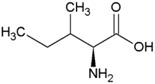	Methionine	Polar, uncharged side chain	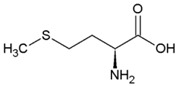
Phenyl-alanine	Hydrophobic side chain	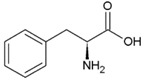	Lysine	Positively charged side chain	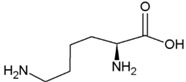
Tyrosine	Hydrophobic side chain	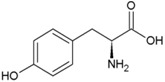	Arginine	Positively charged side chain	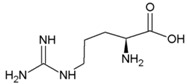
Tryptophan	Hydrophobic side chain	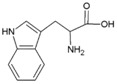	Histidine	Positively charged side chain	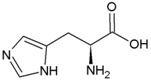
Proline	Hydrophobic side chain	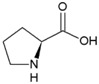	Aspartic acid	Negatively charged side chain	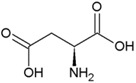
Glycine	Polar, uncharged side chain	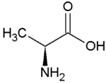	Glutamic acid	Negatively charged side chain	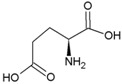
Glutamine	Polar, uncharged side chain	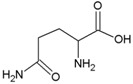	Pyrrolysine	Positively charged side chain	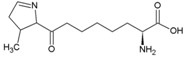
Cysteine	Polar, uncharged side chain	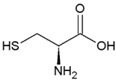	Seleno-Cysteine	Hydrophobic side chain	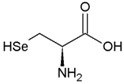

Amino acids have been detected in numerous extreme environments as well as in extraterrestrial meteorites [80,81,82]. Amino acids are rapidly degraded when exposed to UV radiation and other oxidizing conditions [83,84]. However, when buried and protected in the subsurface, they can persist up to 3.5 billion years [25,26]. Due to their relative stability, their high abundance on Earth and their many roles in biological processes, amino acids are considered relevant molecular biomarkers. Their apparent presence throughout the solar system and their chiral properties make these molecules even more interesting.

## 3. Mars and Terrestrial Analogues: Where to Look?

### 3.1. Mars: Past and Present

There are many variables, relating to the geological history of Mars, which influence the probability of detecting traces of life. It is now widely believed that during the first billion year after planetary formation, the environmental conditions on Mars were very similar to those present on the young Earth [85,86,87,88,89]. Recent evidence provided by NASA’s Curiosity rover and Martian meteorites showed that the early atmosphere of Mars had all the prerequisites to develop and maintain life [90,91]. Evaporate minerals, and hydrated silicate minerals (clay minerals), typical for past aqueous activity have been detected on Mars [92,93]. Water may still be present in the subsurface and seep to the surface occasionally [94,95]. These conditions make Mars a prime location for the search for traces of ancient or even extant extraterrestrial life.

Today, the Martian surface is cold (average temperature is −60 °C), dry and hostile and solar ultraviolet (UV) radiation, as well as oxidation processes near the surface are destructive to organic material and life [90,96]. The high UV radiation on Mars is a result of a thin atmosphere (600 Pa) consisting mainly of CO_2_ (95%) [17]. Yen *et al.* [97] speculated that the higher degree of UV radiation generates superoxide ions that would destroy organics at the surface. Indeed, recently, the presence of perchlorates (a superoxide) has been confirmed [98]. The consensus of the scientific community is that drilling into the subsurface and analyzing fresh samples from one to two meter depths will be a prerequisite [99]. Even though the red planet’s current environment is very destructive to organic compounds, there may still be protected environments where life could propagate, or at least traces of ancient life are preserved. Life may be preserved in the subsurface, evaporate deposits, polar regions, northern plains and impact basins and rims [15,100]. Recently detected cave-like structures [101] could represent local environments where life has endured or ancient biomarkers have been preserved.

The effect of Martian extreme conditions on life and its detection can currently only be inferred from research on terrestrial extreme environments. An example is the 2010 paper from Navarro-Gonzalez *et al.* [102] who simulated the Viking-lander GC-MS (Gas Chromatography-Mass Spectroscopy) measurements with Mars-like soil from the Atacama Desert in Chili. The original measurements by the Viking Landers resulted in the detection of chlorohydrocarbons, which were explained to be introduced by terrestrial contamination [103]. However, experiments conducted on Atacama Desert soil samples produced similar molecules in the presence of magnesium perchlorate, a molecule present on the Martian surface [104]. The conclusion was that the formation of the chlorohydrocarbons was due to a chemical reaction between perchlorates and organics present in the soil during heating in the GC oven. These results suggested that organic compounds were present on Mars [102] and show the importance of understanding the processes taking place in Mars analogue environments on Earth.

Although the formation of chlorohydrocarbons can now be largely explained, the source of the organic material remains uncertain. While it is possible that the responsible organics are indigenous to Mars, the current detection of chlorohydrocarbons by Curiosity’s GC as part of “Sample Analysis at Mars” (SAM), which also makes use of thermal volatilization, could also be caused by contamination from terrestrial organics used for derivatization purposes [105]. Partially due to these unforeseen side effects, the presence, or absence of organic molecules on Mars remains to be proven.

### 3.2. Terrestrial Extreme Environments

There is a wide variety of extreme environments on Earth that display Mars-like conditions. However, no environment on Earth exists that displays all factors present on Mars simultaneously. Only with the use of artificial simulation chambers, a more defined approximation of the Martian environment can be achieved. Conditions like pressure, CO_2_ concentration, temperature, radiation and humidity can be closely regulated in a sealed off system and, thus, the effects of specific selected environmental parameters on life and its components can be investigated. This approach has been used to assess viability of a variety of terrestrial microorganisms under Martian conditions [106,107], but also to determine if methane production by methanogenic archaea could theoretically occur in the Martian surface [108]. However, much is still to be learned by investigating life in extreme Mars-like environments on Earth, where live had billions of years to evolve, a situation that obviously cannot be mimicked in short-term Mars simulation.

On Earth, there are various parameters determining the chances of detecting life and biomarkers in extreme environments. Several environments are discussed below and although they differ substantially, a general tendency is seen in how microorganisms adapt to these hostile environments in which minerals appear to play a crucial role. Sulfated rock types like Halite or Gypsum have been shown to protect microorganisms from desiccation and UV irradiation while at the same time they still allow for processes like photosynthesis and nitrogen fixation due to electron cycling [109,110]. Interactions between microbes and such minerals provide the possibility for life to seek shelter from surface conditions, which could also be the case for Mars.

#### 3.2.1. Hot Deserts

Deserts and other hyper arid areas are amongst the most isolated places on Earth. Constant exposure to sunlight and thus high UV-radiation, desiccated conditions, high temperature and high salinity due to low water availability make these environments deadly to all but the most hardy organisms. In these driest places, we often find low quantities of organic compounds [29,82,111]. However, they are also frequently below the detection level of the employed instruments [82,112,113]. Thus, desert areas appear very patchy, making it challenging to localize microbial life or preserved organics [114]. Nonetheless, extremophilic microbes have been detected in desert surface samples albeit in low quantities; large varieties were detected in samples taken within geological proximity [114]. Studies by Carson *et al.* previously suggested that mineral type [34] and soil connectivity [115] can have a strong influence on the microbial diversity, offering an explanation for the observed varieties over a small geological scale.

Although the desert surface contains only limited traces of life, the subsurface or the inner matrix of rock formations sometimes shows a completely different picture. Parro *et al.* [116] described a deposition at two meter beneath the barren surface of the Atacama Desert consisting of halite-, nitrate- and perchlorate-containing salts where a variety of microorganisms was identified. Gypsum containing rock formations in the Tunisian Sahara desert sustained a microbial habitat just beneath the surface of the rock, where the microorganisms were protected from damaging UV radiation and thin liquid water films could be provided by adsorption to the mineral matrix [117]. These are just two of many examples suggesting that life in deserts localizes and specializes towards areas that offer protection from the harsh UV radiation, and desiccated conditions, throughout (and underneath) the open planes.

#### 3.2.2. Subsurface Environments 

Complete ecosystems exist underneath the Earth’s crust with readily available energy supply [118], which can also be extrapolated to Mars where surface conditions are too extreme to maintain life. Microorganisms that live in subterranean habitats depend on traces of reduced inorganic compounds such as sulfur, hydrogen and iron for their energy supply, which are provided by minerals [34,119], and can maintain a community for as long as sources of these compounds remain bioavalaible [120]. Deep caves, and in particular subsurface mines provide relatively good accessibility to subsurface communities and novel species.

One of the most striking examples of self-sustaining subsurface environment is Movile Cave, Romania. This cave system has been isolated from Earth’s atmosphere and sunlight for 5.5 million years and displays a unique groundwater ecosystem, which is supported by *in situ* chemoautrophic production [121,122]. The cave’s atmosphere is rich in hydrogen sulfide, carbon dioxide and also contains 1%–2% methane and as a result chemosynthesis is mainly based on sulfide- and methane-oxidation [23,123]. Although this cave has been deprived of sunlight for millions of years, a variety of unique indigenous species have been identified, including 33 vertebrates [121] and a wide range of microorganisms, ranging from common alpha-proteobacteria to methanotrophs [124].

The Boulby Mine, which is located at 1.1 km depth on the northeast coast of England, displays an environment with high salinity, low water availability and the presence of 250 Ma (million years) old halite- and sulphate-salts, which provide acceptors for electron transport [125,126]. The presence of anaerobic halophilic microorganisms in its brines displays the possibility for sustained life in environments that have been cut off from the atmosphere. Microbial life was also detected in the 2.7-billion-year-old Ventersdorp Supergroup metabasalt, located underneath the Mponeng Gold mine in South Africa at 3 to 4 km depth. This mine displays brine systems dominated by bacteria that obtain their energy from hydrogen oxidation linked to sulfate reduction [20]. High pressure and salinity keep water in an extended liquid state [127] and microorganisms have been shown to be adaptable to these circumstances [128], indicating that such ecosystems may in theory exist underneath the frozen surface of Mars as well.

#### 3.2.3. Polar Region: Antarctica

The McMurdo dry valleys of Antarctica are among the harshest environments on Earth. Mean temperatures of –20 °C, low water availability, high solar radiation, desiccating winds, diurnal freeze and thaw cycles and low light intensity create an environment to which microorganisms do not adapt readily [129]. The McMurdo dry valleys are the only known locations on Earth that contain dry permafrost, a condition also present in the Martian arctic regions. Furthermore, the temperature above 1500 meters does not exceed 0 °C and thus liquid water availability is overall low. The McMurdo Dry Valleys encompass an area of 15,000 km^2^ and approximately 30% of its area is free of ice and snow on the surface but large deposits of solid frozen water exists under the surface of these regions [130].

Biomass is low in these regions but just like in other extreme environments, life tends to seek for shelter in areas that provide protection against the most destructive effects of desiccation and radiation. Sub-glacier depositions and subsurface lakes covered with thick ice crusts, offer strongholds, protected from the most damaging conditions. High pressures, and high salinity of water reservoirs or brines running through subsurface or ice depositions lowers the freezing point of water and thus provides liquid water in temperatures as low as –50 °C for biochemical processes [127,131]. Besides liquid water, an energy source (e.g., reduced compounds or sunlight) must be at hand to sustain biotic life. There are indications that large frozen bodies of water exist on Mars [132], and also several of the Jovian moons contain an ocean of water underneath a thick ice cover or sandwiched between ice layers [133,134,135,136]. These discoveries highlight the importance of investigating and understanding these types of environments.

**Figure 4:**
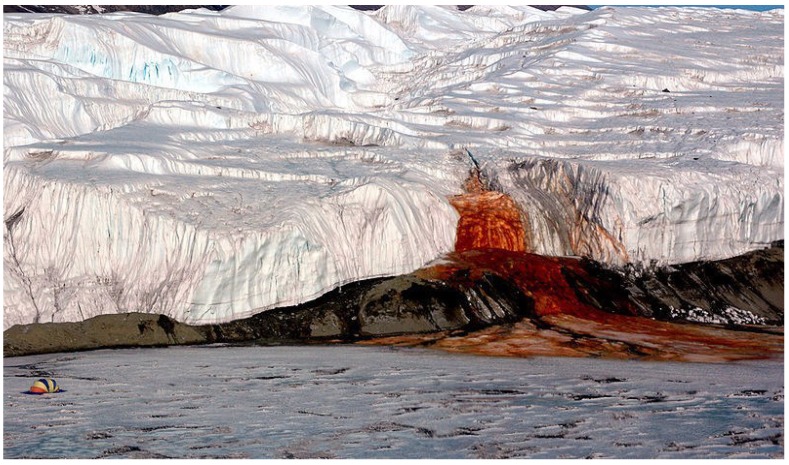
Blood falls glacier owns its distinctive red color due to high ferrous iron concentrations (CREDIT: United States Antarctic Photo Library).

The Blood falls (Figure 4), an iron-rich subglacial outflow from the Taylor glacier, has been secluded from the atmosphere for at least 1.5 million years. It maintains a microbial community which cycles sulfur in a sulfate-rich ancient marine brine with ferric iron as the final electron acceptor [137]. This specialized metabolic pathway results from the low carbon load that in turn is a direct result from low levels of photosynthesis, creating an anaerobic, ferrous iron-rich environment. The high amount of iron gives the glacier its distinct blood-like red color.

In addition, the brine encapsulated underneath the permanent ice cover of the Antarctic lake Vida maintains an active ecosystem at –13 °C. This is an anoxic, slightly acidic (pH 6.2) environment where the brine is dominated by sodium chloride, which makes it extremely saline (salinity estimated at 245, practical salinity scale) [138]. The brine system is dominated by metabolically active and phylogenetically diverse bacteria that live in the presence of high levels of ammonia, molecular hydrogen, dissolved organic carbon, reduced metals and oxidized species of sulfur and nitrogen [132]. This ecosystem has been isolated from external sources of energy for almost 3000 years [138], indicating the presence of other long-term internal energy reserves. The geochemistry of this brine suggests that abiotic brine-mineral interactions play a role in the processes of creating a rich source of dissolved electron acceptors, which makes methanogenesis and sulfate reduction energetically unfavorable [132].

Another Antarctic lake, lake Vostok, has been secluded from the atmosphere and sunlight for over a million years [139]. The presence of a wide variety of microorganism sequences in the 3700 meter thick ice layer has been revealed. Over 3500 unique sequences specific for aerobic, anaerobic, alkaliphilic, thermophilic, psychrophilic, halophilic, acidophilic, desiccation-resistant, autotrophic and heterotrophic organisms have been detected. Bacteria, archaea, eukarya, and even some multicellular eukaryotes were detected [22]. Microorganisms that are active in many phases of the nitrogen cycle as well as those that fix, utilize and recycle carbon were detected. In addition, the activity, determined by RNA assays, and the count of microorganisms present near the embayment of the accretion ice, compared to the overlaying meteoric ice was higher. Therefore, Shtarkman *et al.* [22] concluded that viable and active microorganisms may also be propagating in the lake water underneath and that geothermal activity may be the sole energy provider next to energy-rich compounds.

### 3.3. C-Type Meteorites

Meteorites can easily be considered extreme environments. Since their formation, meteorites have been exposed to the vacuum of space and have been bombarded by solar radiation. Impact events as well as entry through a planet’s atmosphere generate extremely high temperatures, capable of destroying organics. However, inspection of several carbonaceous (C-type) meteorites revealed the presence of low-temperature mineral assemblages that have not been extensively heated (<100 °C), which has been explained by the presence of pre-impact fluid inclusions in meteorites that could offer organics extra protection against excessive heating [140,141]. Fragmentation of a meteorite prior to impact, as observed in 1965 over Revelstroke, Canada, also produces fractions of less heated meteorite, protecting organics from the most cataclysmic events of impact [142]. Still, meteorites, or their parent bodies are hardly potential habitats for extant life. Claims of fossilized extraterrestrial microbial life detected on meteorites [143,144,145] are substantial claims and, therefore, need substantial evidence, which has, thus, far not been provided.

Nonetheless, meteorites are the only source of extraterrestrial organic compounds that have been analyzed to date and are deemed vital to increase our understanding of interstellar chemistry. Many of these meteorites have been adrift since the formation of our solar system and, thus, may contain chemical compounds that predate biotic life [68,80,81,82]. The carbonaceous chondrites (C-type meteorites) comprise a class of meteorites that contain ~2% weight of organic carbon [146] and hit the surface of Earth on a regular basis. The Murchison meteorite, which has been subject to detailed scientific research, contained at least 66 amino acids, N-heterocycles, carboxylic acids, sulfonic and phosphonic acids, and aliphatic and aromatic hydrocarbons [50,51,81,146,147,148,149]. Impacts of meteorites can spread these types of molecules on a planet’s surface, creating organic-rich niches and, thus, facilitate the presence of at least one of the prerequisites to the formation of life. Formation of these organic molecules is believed to arise due to aqueous processes on the parent bodies (asteroids and comets) [150]. Recent findings indicate there may also be other mechanisms that lead to the formation/alteration of organic compounds detected in meteorites, namely during impact [6,151]. However, the identification of a wide range of organic compounds, including several amino acids, on the comet 81P/Wild 2 [152,153] suggests that at least part of the organic inventory is present prior to impact.

The investigation of meteorites has yielded important scientific insight and will most likely continue to do so the coming decades. The observation that organic molecules survive the violent journey through space suggests that the detection of organics on other planets like Mars is also within reach. The Antarctic Search for Meteorites (ANSMET) [154] is continuously scanning the Antarctic planes for meteorites, which are relatively easily detected on the snowy white surface of Antarctica.

## 4. Techniques Currently in Use for Biomarker Detection: How to Look Here?

Life detection techniques generally comprise the extraction of biomolecules followed by an analytical component, such as High Performance Liquid Chromatography (HPLC) or Gas Chromatography (GC) coupled to Mass Spectrometry (MS) or fluorometric detection. Microarrays and immunoassays are also used to identify biomarkers. While genomic material can be multiplied using the Polymerase Chain Reaction (PCR), many other biomolecules are not prone to such multiplication techniques and thus need to be concentrated for detection and identification. Biomolecules or biota can be freely present in water reservoirs but can also be intercalated in the matrix of minerals, which obstructs efficient extraction [30,31,32,155,156].

Efficient extraction of biomarkers from these minerals is the crucial step in detection because many minerals show high potential for the long-term preservation of biomolecules [157,158,159]. In order to minimize biases in extraction due to soil particle size, a universal sample preparation method, which may include crushing, grinding or milling of the sample, has been proposed by Beaty *et al.* [160].

One approach to enhance biomarker extraction is acid digestion, aimed at dissolving the entrapping mineral, and releasing the biomolecules from salt deposits like sulfates or other evaporate minerals [161]. These sulfate-rich evaporates may be especially important with regard to the detection of life since microbial sulfate reduction is implicated in rocks that contain the oldest known traces of life dating 3.5 billion years back [162]. Hydrofluoric acid has also been used to dissolve minerals and increase extraction yield, but was shown to have destructive effects on more fragile biomolecules such as DNA [30]. However, other biomarkers may be more resistant to such an aggressive method, as was observed for proteins that were recovered from minerals by a similar approach [163].

More commonly, a wide range of solvents is used to extract organics from their environment. Aqueous polar solvents are used to extract the polar substances (e.g., amino acids, DNA) and organic solvents are used to extract the non-polar organics (e.g., hydrocarbons, lipids). Solvents can be adjusted to improve extraction. Direito *et al.* [30] used a hot phosphate-rich ethanolic buffer in the cell lysing step during DNA extraction from soil samples, reasoning that the phosphate would compete with the phosphate backbone of DNA for chemical binding to the soil matrix. This resulted in up to a hundredfold higher recovery of DNA. Such mechanism-based improvement of extraction could potentially also be used to enhance extraction of other biomarkers.

The use of surfactants in combination with aqueous solvents to apply combined solving power for polar and non-polar compounds is another good example of optimization of extraction/detection techniques [164,165,166]. Surfactants are amphiphilic molecules, which makes them capable to interact with hydrophilic as well as with lipophilic compounds [167]. The possibility to obtain both polar and non-polar molecules in one extraction is a large advantage over other extraction techniques and the exclusion of aggressive solvents like methanol or acetonitrile permits coupling to sensitive antibody-based detection techniques, as was planned for the Life Marker Chip (LMC), which was initially intended for ESA’s ExoMars mission [164,165]. However, the extraction yield of hydrophobic molecules by surfactant-based aqueous solvents only displayed about one third of the extraction rates observed when using organic solvents such as methanol-based solvents [165].

An alternative and attractive approach for the extraction of biomarkers with different polarities from a sample is offered by “subcritical water extraction”. This technique uses water as an extraction solvent at temperatures between 100 °C and 374 °C, while maintaining it liquid under high pressure (up to 22 MPa). These conditions dramatically alter the dielectric constant ε (*i.e.*, the polarity) of water molecules: water at ambient temperature and pressure has a ε = 79 while increasing the water’s temperature to 250 °C and 5 Mpa yields ε = 27 [168], which is similar to ethanol at 25 °C and 0.1 MPa. By changing temperature and pressure it is possible to extract a range of polar as well as non-polar molecules with high efficiency [169,170,171], while the eventual extracts will be dissolved in water, making follow up applications less dependent on the solvent type, which would be appropriate for antibody based assays like the LMC.

The principle of a microarray/immunoassay approach shows great prospect in the field of life detection since this approach allows for screening many biomarkers at once by aiming for a multitude of generic molecular structures or, if preferred, very specific structures or sequences [166]. Another major advantage of using an antibody based detection instrument is that samples do not need to be heated prior to analysis. This eliminates the danger of perchlorates, present in the Martian soil, destroying, or altering organic matter under heated conditions. The SOLID instruments (Signs of Life Detector), of which SOLID3 is the latest, are microarray-based instruments designed for astrobiological purposes and can detect a wide range of molecular compounds ranging from peptides to whole cells and spores [172,173]. Extraction of biomolecules from the soil is conducted by using a water-based extraction buffer containing 0.1% surfactant (Tween 20), combined with sonication steps [172]. Relatively cheap reproduction of microchips and the possibility of reuse by washing with an eluting solvent makes them very suitable for astrobiology missions, with solvent carrying capacity being the major limiting step. However, no extensive testing of the functionality of antibodies in open space has been done to our knowledge and the effects of such an environment on the functioning of antibodies needs to be investigated.

Other approaches for the detection and identification of biomarkers are often based on the separation of molecules by their binding and solving behavior with respect to a stationary adsorbing phase (column) and mobile solving phase (solvent). This is the basic principle of separation techniques such as High Performance Liquid Chromatography (HPLC) [174]. Due to differences in binding and solving characteristics, some molecules are retained longer on the column than others, providing a means of separation and thus identification. Detection most often occurs via UV-visible light, fluorescence-detection or mass spectroscopy [175], where in the latter case the mass-to-charge ratio of ionized molecule fragments is determined as a means of identification, resulting in very high sensitivity [176]. To improve separation and fluorescence detection, derivatization methods have been developed, which are often used in amino acid analyses [177,178] and can also improve separation of amino acid enantiomers [179,180]. Gas chromatography is also based on separation of compounds by retention to a stationary phase but utilizes a carrier gas instead of a liquid mobile phase, and the column is contained in an oven to regulate the temperature of the gasses, of which the vapor pressure is used to determine the concentration of gaseous analytes [181].

Methods for the identification of microbial communities are often based on *in situ* extraction of genomic DNA from samples rather than by a culture based approach [182,183]. As a consequence, the yield of genomic material is relatively low, and an underestimation of the microbial diversity can occur through interspecies differences in extractability or proneness to amplification techniques [184]. There are several approaches to perform the cell lysing step, but currently the bead-beating methods are considered to give most satisfying results [185]. After extraction, DNA can be amplified by PCR [49]. The most used approach in identifying microbial communities is to screen for specific gene segments, such as 16S ribosomal RNA (rRNA) gene segments [39], which is present in all terrestrial microorganisms but expresses slight differences in their sequence per species, by which they can be related to other species. An approach aiming to amplify conserved terrestrial sequences may however not be adequate in the search for extraterrestrial life [39]. Even on Earth, screening for the 16S rRNA gene does not detect all organisms [186]. Whole genome amplification (WGA) may be a better approach if the goal is to detect unknown life that utilizes DNA-like molecules. Microbial communities have been identified with this technique that would not have been identifiable with a specific primer based approach [187]. The use of primer libraries, to cover a wide variety of amplifiable DNA gene sequences, would also increase the chance of identifying otherwise undetected species. Furthermore, with the arrival of next generation sequencing techniques it has become possible to obtain huge amounts of sequences in a massive parallel reaction in a fraction of the time (and costs) that Sanger-based sequencing would require [188]. A perquisite for detection of extraterrestrial DNA however would still be a partial overlapping evolution, or an identical genesis of DNA-based life, of which the former possibility seems more likely than the latter due to the complexity of the molecule.

In summary, the availability of a large variety of approaches is advantageous to life detection. Combining various techniques, for example different extraction and detection techniques on the same sample allows us to obtain robust and comprehensive results. Detection and correlation of a variety of biomarker classes would induce more diagnostic power than the characterization of just a single biomarker class. For example, Lester *et al.* [111] combined total organic carbon (TOC) load and phospholipid fatty acids (PLFA) analysis data with DNA profiles. Such combined approaches can greatly increase our knowledge of the interactions between biota, biomarkers and the environment.

## 5. Current Instrumentation on Mars Life Detection Missions: How to Look There?

Current and future Mars missions will search for past and present life with the help of instruments that can identify traces of life. Organic molecules; microfossils or isotopic data indicating microbial activity in rocks are all tracers for biological processes. Life detection instruments are optimized to detect robust biomarkers such as (homochiral) amino acids and small hydrocarbons [14,48]. The Sample Analysis at Mars (SAM) instrument on the NASA Curiosity rover is currently operating on Mars. SAM is equipped with three instruments that aim to detect organic compounds: A Quadrupole Mass Spectrometer (QMS); a Gas Chromatograph (GC); and a Tunable Laser Spectrometer (TLS). GC-MS analysis can be performed by combining the two instruments, which facilitates separation on the GC column and subsequent identification of organic compounds by the MS component. The TLS gathers isotope ratios for carbon and oxygen from carbon dioxide and can measure trace levels of methane and its carbon isotope [189]. Other instruments, which include CheMin (Chemistry and Mineralogy) and RAD (Radiation Assessment Detector), are also incorporated in the Curiosity rover and can identify mineral types by X-ray diffraction or measure radiation, respectively. A recent discovery of Curiosity rover is the identification of an environment at Gale crater, which was once an aqueous environment with neutral pH, low salinity and variable redox states for both iron and sulfur, capable of sustaining life on a chemolitoautotrophic basis [190]. Water was also detected, bound within the amorphous soil components [191].

The Pasteur payload for the joint ESA-Roscosmos Exomars mission in 2018 has been revised several times. The Mars Organic Molecular Analyzer (MOMA) is the most powerful organics-detection payload instrument, which can detect molecules in the range of ppb-ppt [29]. This instrument uses a laser to combust organic materials, after which the resulting products will be separated by GC and identified with ion-trap coupled mass spectroscopy [192]. The laser used in the GC is also able to combust compounds ranging from volatile to non-volatile compounds, thereby increasing the detection range. A GC column is specifically designed for detection of homochirality in amino acids. Another important function embedded in MOMA is the direct derivatization of organic compounds with labile hydrogen groups (e.g., amino acids, nucleobases) to stabilize them and improve separation detection sensitivity. A wide range of biomarkers can be detected, increasing the chance of finding traces of life (if ever present). By implementing thermal volatilization for the GC, a drawback of MOMA could be the reaction induced by a combination of organics and perchlorates under heating, resulting in the production of chlorohydrocarbons [102]. Other instruments on the ExoMars payload include a Raman spectrometer, an infrared imaging spectrometer. Spectrometric detection techniques like Raman spectroscopy are non-destructive and independent of consumables like extraction solvents and rinsing buffers. Another advantage is that sample processing is not required. However, most promising is that it can measure biological and geological signatures simultaneously. These characteristics make Raman spectroscopy ideal for robotic remote field missions [193], and thus a valuable asset for astrobiology, especially as a tool complementary to more analytical instruments like GC or microarrays. Several instruments have been de-scoped, such as the LMC and the Urey instrument. The latter is equipped with a Subcritical Water Extractor (SCWE) and its main goal is to determine biotic *versus* abiotic origin of detected molecules, such as amino acids and nucleobases but also targets PAHs [194].

## 6. Conclusions and Looking forward

We have provided an overview of biomarkers and techniques relevant for life detection and what knowledge can be obtained by investigating extreme environments on Earth as a template for Mars. A large variety of biomarkers are available for tracking past or present life. Although life uses and produces many different biomolecules, not all are suitable as diagnostic tools. Preservation potential and extractability of a molecule are parameters that must be taken into account. In addition, the original concentration as well as the spatial distribution of biomarkers will influence the chances of detection. Furthermore, a distinction must be made between extant and extinct life. Most of the molecular compounds that are typical for extant life (DNA, carbohydrates, ATP, proteins) have a relatively short lifetime and, thus, degrade fast outside the protected confinements of a cell, especially when exposed to UV radiation or oxidizing conditions present on Mars. Robust biomarkers would form a more appropriate target for extinct life.

If extraterrestrial life exists, it likely also makes use of membrane compartmentalization [55]. Lipids are therefore considered high priority targets [14,48]. Amino acid enantiomeric excesses are often used as proof for biotic origin [77]. However, the uncovering of abiotic processes that could lead to homochirality in amino acid mixtures has made enantiomeric excesses lose some of its diagnostic power for biotic origin. Combined approaches may be needed to explain enantiomeric excess rather than using it as a diagnostic means by itself. Targeting of nucleic acids with non-specific amplification techniques such as WGA may reveal the existence of alternative forms of DNA [195], and the development of next generation sequencing techniques will most likely have a large impact on the search for extraterrestrial life. Scanning for “hypothetical biopolymers” by using synthetically created molecular probes could potentially reveal the presence of alternative nucleic acids.

Investigation of extreme environments on Earth has shown us that microbial life tends to prefer relatively sheltered places. Life and biomolecules accumulate in these environments that offer protection against desiccation, UV radiation and other degrading effects. If there is, or was life on Mars, one might expect the same. This implies a strong focus towards localizing potentially protective environments on Mars. Rock and ice formations, caves and subsurface brine systems would be good candidates to look for traces of life. The role of clay-rich minerals in these local areas is important since they offer the desired protection and can function as a catalyst for chemical reactions. Aiming for such clay-rich formations is, therefore, given consideration in current life detection strategies. However, the adsorbing properties of clay rich minerals in turn may hinder efficient extraction of biomolecules. An important strategy for future life detection techniques should be the optimization of biomolecule extraction from clay-rich minerals. A good approach is to combine solvents with a competitive binding molecule that would free biomolecules from the mineral surfaces. Switching between polar and non-polar solvents, the addition of surfactants or using pressurized solvent extractions are other approaches to increase extraction rates and should be further optimized.

The identification of extracted compounds can be conducted by many different techniques. Since many biomarkers demand specific procedures to be identified it is a complex endeavor to analyze soils for their contents with a single procedure. Separation and detection in techniques, such as HPLC or GC, often require special derivatization protocols that are not universally applicable to different biomolecules. A future goal would be to develop a “standard life detection package” for use in astrobiological missions. Immunoassays and microarrays may be of specific use in this context. These assays can identify thousands of compounds by binding or hybridizing to specifically created antibodies or DNA probes (lock-and-key mechanism). Immunoassays, specifically designed to detect a range of biomolecules indicative for extant or extinct live could facilitate an easy read-out for sample analysis. Examples of such approaches include the LMC and the SOLID instruments discussed above and are gaining in popularity. Improving compatibilities between antibodies and the more aggressive organic solvents may be necessary in order to make this assay more effective for the detection of non-polar compounds, such as PAHs, lipids and pigments, which are more efficiently extracted with organic solvents. The concentration of methanol has been shown to effect the formation of immune complexes [196]. Alternative affinity tools based on nucleic acids (aptamers), polypeptides (engineered binding proteins) and inorganic molecular imprinted polymers can be selectively produced to have higher chemical and physical stability, which would be a major advantage if a more hazardous extraction solvent is to be used [197].

It remains important for future planetary missions to search for robust biomarkers in regions with high organic preservation potential. The optimization of extraction methods that could extract polar and non-polar molecules from clay-rich mineral samples is equally important. A special focus to advance techniques that can identify many different biomarkers at once would be a major contribution to future planetary missions.
